# Is lifestyle Modification the Key to Counter Chronic Diseases?

**DOI:** 10.3390/nu14153007

**Published:** 2022-07-22

**Authors:** Panagiota Mitrou

**Affiliations:** Independent Department of Therapeutic Protocols and Patient Registers, Hellenic Ministry of Health, 104 33 Athens, Greece; panagiotamitrou@gmail.com

Dietary patterns, defined as the quantities, proportions, variety, or combination of different foods and drinks, as well as the frequency with which they are habitually consumed, are associated with an increased or decreased incidence of chronic diseases. Several lines of evidence indicate that healthy diet and exercise can prevent cardiovascular diseases, stroke, diabetes, and some types of cancer. Lately, an association has been found between eating habits, exercise, and psychological and/or mental disorders.

In the first article of this Special Issue, Gantenbein and Kanaka-Gantenbein reviewed the beneficial effects of the Mediterranean Diet and specifically its antioxidant and anti-inflammatory properties, enhancing metabolic, reproductive, and mental health [1]. 

As an alternative, the ketogenic diet, based on carbohydrate restriction, moderate protein intake, and increased fat consumption, has gained ground [2]. The restriction of carbohydrates results in an increase in the production of ketone bodies, a metabolic state in which the body utilizes fat (instead of glucose) as its primary metabolic substrate. According to Dowis and Banga, the use of the ketogenic diet might have many therapeutic effects, including weight reduction and an improvement in predisposing factors for cardiovascular diseases such as dyslipidemia and hyperglycemia [2].

However, if one finds a low-carbohydrate ketogenic diet difficult to comply with, then targeting the microbiome might be a way to improve metabolic health. According to Turroni et al., dietary products based on symbiotic agriculture could possibly modulate the human microbiome and metabolome, decreasing the risk of metabolic syndrome [3]. Moreover, as stated by van Krimpen et al., reducing gut permeability via *Lactobacillus* spp. supplementation could be a potential treatment to reduce cachexia, an inflammation-driven condition related to aging and chronic diseases [4].

Among other beneficial effects, healthy nutrition appears to have an impact on inflammatory and rheumatic diseases. As highlighted by Ratajczak et al., the type of diet followed is particularly important for patients suffering from Inflammatory Bowel Disease (IBD) who often display vitamin deficiency, such as low folate levels, resulting in anemia, neurological symptoms, and bone loss [5]. In these patients, although well-balanced dietary patterns are needed to provide all macro- and micronutrients, fiber-rich products often exacerbate gastrointestinal discomfort. As a result, the concentration of folic acid should be often evaluated in patients with IBD and given as a supplement in cases of insufficient dietary intake and/or absorption [5]. Basdeki et al. performed a systematic review and meta-analysis of previously published randomized controlled trials (RCTs) investigating the relationship of sodium intake with systemic inflammation [6]. Although the study did not verify the hypothesis that sodium intake can induce a systemic inflammatory response in humans in a dose–response manner, it highlighted major issues that could be addressed in future relevant RCTs [6]. In a systematic review, Tsiogkas et al. summarized the current evidence regarding the efficacy of Crocus sativus (Saffron) supplementation in patients with rheumatic diseases (RDs) [7]. Although current evidence indicates that saffron may have a positive effect in patients with RD, targeting inflammatory and immune responses, oxidative stress, pain, and the psychological effects of the disease, more studies are required to shed light on the efficacy and draw conclusions on the appropriate dose of this supplement [7]. 

In the present Special Issue, several articles focus on the effects of nutritional interventions on cardiovascular risk factors such as diabetes, hypertension, and increased body weight. According to Mahrouseh et al., eating vegetables several times a week in addition to performing physical exercise may reduce the risk of DM, although more lifestyle characteristics and socioeconomic conditions possibly underlie the increased prevalence of diabetes in Slovakia and other European countries [8]. The association between high fructose consumption and hypertension is controversial. Based on the study by Béghin et al., although the consumption of increased quantities of fructose derived from non-natural foods is associated with elevated diastolic blood pressure, the consumption of natural foods containing fructose, such as fresh fruits, does not increase blood pressure and thus remains a healthy dietary habit with pleiotropic positive metabolic effects [9]. The findings of Awoke et al. showed that women across reproductive life stages, in particular those with lower socioeconomic status, often fail to meet dietary and physical activity recommendations and should be targeted in future interventions to prevent weight gain [10]. Familial socioeconomic disadvantage was also identified as a common risk factor for obesity and early-childhood caries, which are two highly prevalent chronic diseases in childhood. In the above-mentioned study by Manohar et al., children with the highest trajectories of discretionary food intake were more likely to have increased body weight, highlighting the need for targeted health promotion interventions [11]. As reviewed by Pereira et., most dietary interventions to reduce childhood obesity are based on person-based educational approaches with modest (if any) effects on body weight, highlighting the need for multilevel interventions focused on micro- and macro-policy environmental changes and the strengthening of children, family, and community [12]. From this point of view, Scazzocchio et al., presented a new Italian educational program developed for Italian students, aiming to increase food literacy and favoring a healthier relationship with food [13]. 

Dietary choices may also have an impact on neurological diseases and mental health. As described by Wang et al., unhealthy dietary choices, such as the excessive intake of saturated fats and salt, can affect brain function and increase the risk for ischemic stroke [14]. On the other hand, as stated by Stoiloudis et al., healthy nutrition shows promising beneficial effects in terms of slowing down multiple sclerosis activity and progression [15]. Moreover, healthy nutrition may protect against anxiety and psychosocial maladjustment, as shown by Freret et al. in animal studies and by Khaled et al. in human studies [16,17].

Healthy nutrition can also help enhance athletic performance. In a cross-sectional study conducted by Martínez-Rodríguez et al. in Spanish female athletes, a correlation analysis highlighted the relationship of increased body weight and poor nutrition with worse results in power and endurance tests. As expected, exercise had also a beneficial effect on bone density [18]. Based on the study by Kyle et al., increased physical fitness and aerobic exercise are also important for women across the first postpartum year experiencing changes in bone mineral density and serum lipids [19].

Given the increasing prevalence of diet-related chronic diseases, in the last paper of this Special Issue, Wu et al. highlighted the need for the development of new scalable dietary monitoring techniques and presented the results of a study in Switzerland in which the nutritional quality of users’ diets was estimated based on digital receipts from grocery shopping [20]. 

During the last few decades, there has been a tremendous rise in the incidence of chronic non-communicable diseases (NCDs) such as cardiovascular diseases, cancer, diabetes, and chronic respiratory diseases. Although population aging and other unmodifiable genetic factors are contributing to this rise, it has been recognized that there also modifiable, unhealthy lifestyle choices, such as a diet rich in saturated fats, salt, and refined carbohydrates, sugar-sweetened beverages, a lack of physical activity, and smoking, which exacerbate this phenomenon [1]. Identifying the relationship between lifestyle choices and chronic diseases can help stakeholders to design and implement programs that are oriented toward a healthy lifestyle, ensuring progress towards achieving the goal of reducing premature deaths from NCDs by one third by 2030, as outlined in the United Nation’s 2030 Agenda for Sustainable Development. 
